# Determination of a Screening Metric for High Diversity DNA Libraries

**DOI:** 10.1371/journal.pone.0167088

**Published:** 2016-12-08

**Authors:** Nicholas J. Guido, Steven Handerson, Elaine M. Joseph, Devin Leake, Li A. Kung

**Affiliations:** Gen9 Inc., Cambridge, Massachusetts, United States of America; Seoul National University College of Medicine, REPUBLIC OF KOREA

## Abstract

The fields of antibody engineering, enzyme optimization and pathway construction rely increasingly on screening complex variant DNA libraries. These highly diverse libraries allow researchers to sample a maximized sequence space; and therefore, more rapidly identify proteins with significantly improved activity. The current state of the art in synthetic biology allows for libraries with billions of variants, pushing the limits of researchers’ ability to qualify libraries for screening by measuring the traditional quality metrics of fidelity and diversity of variants. Instead, when screening variant libraries, researchers typically use a generic, and often insufficient, oversampling rate based on a common rule-of-thumb. We have developed methods to calculate a library-specific oversampling metric, based on fidelity, diversity, and representation of variants, which informs researchers, prior to screening the library, of the amount of oversampling required to ensure that the desired fraction of variant molecules will be sampled. To derive this oversampling metric, we developed a novel alignment tool to efficiently measure frequency counts of individual nucleotide variant positions using next-generation sequencing data. Next, we apply a method based on the “coupon collector” probability theory to construct a curve of upper bound estimates of the sampling size required for any desired variant coverage. The calculated oversampling metric will guide researchers to maximize their efficiency in using highly variant libraries.

## Introduction

Recent advancements in DNA synthesis and assembly techniques have enabled the production of highly diverse libraries with relatively even distribution of variants [1–5]. These synthetic DNA libraries allow the sequence space of antibodies, enzymes, various other proteins, and genomes to be more thoroughly examined [6–9]. An example of the use of a DNA library in antibody research is the screen of a library of 10^10^ variants for the humanization of antibodies [10]. Such antibody libraries, typically have 2–3 amino acid possibilities at each variant codon position in the complementarity-determining regions. The large diversity of such a library facilitates the discovery of antibodies with desired properties (e.g. humanized).

It is paramount when screening a DNA library, to efficiently use resources to test a large percentage of the variants represented. In order to determine the appropriate amount of screening to conduct, it is important to take into account the fidelity and diversity of the library along with the representation of the library variants. We define fidelity as a measurement of the fraction of library members lacking errors (insertions, deletions, substitutions or rearrangements). Diversity is defined as the number of different library members (distinct variants) present in the library population. And representation is how closely the relative frequency of all distinct variants matches the intended distribution.

To measure and evaluate these DNA library metrics, next generation sequencing (NGS) will be applied. Next generation sequencing is the current state of the art for measuring large numbers of individual DNA sequences. Even with the recent advances in NGS, it remains difficult to directly measure the representation of variant libraries, as the number of reads is insufficient to cover the size of a large library. As an example, a 1 kbp combinatorial DNA library with a billion variants has equivalent base pair content to that of 300 human genomes. Thus, brute force measurements of individual library members is impractical even with field-leading sequencing capabilities; i.e. >300 million reads at ~150 base lengths.

A more informative indicator of library quality is the degree of oversampling required for the screening of a given DNA library. A measure of oversampling not only describes how well a library covers the intended sequence space but also takes into account the traditional metrics of fidelity and diversity as well as extending the variant coverage metrics to include representation. In all, an oversampling metric provides a practical, statistical approximation describing the number of molecules to be screened and ensuring that a desired fraction of the members of the library are interrogated.

An oversampling metric is derived from the distribution frequency of individual variant positions within the library. However, measurement of these distributions using next generation sequencing requires accurate mapping of individual reads to their respective references. Achieving alignment accuracy is a technical hurdle as recent methods to speed up alignment processing of NGS data for genomic applications rely on seeds (matching k-mers) and heuristics; and therefore do not guarantee that the alignment found is optimal. Variant libraries, with many similar but different member sequences, are not well suited to seeded methods. To overcome this challenge, we have developed a novel computational method, which more efficiently and accurately aligns the sequencing data from variant libraries. With these accurate alignments, we produce the frequency distributions that are the basis for understanding fidelity, diversity, and representation of libraries.

Furthermore, we have extended the statistical “coupon collector” problem[11–13] to use the frequency distributions to generate a library oversampling metric. The “coupon collector” problem considers the number of picks necessary to collect at least one of each type of coupon from independent picks of a given distribution of coupons. The answer for the case of uniform coupon probabilities has a relatively simple and exact combinatorial solution, based on the expected number of picks to collect the next unseen coupon in a series of picks.

For the case of a non-uniform coupon distribution, it is possible to describe a full collection, with a simple expression, which can be used with numerical integration to get an exact theoretical expectation:

$$\int_{t=1}^{\infty }1-\prod_{i}^{}1-e^{-t\lambda_{i}}$$

where t is the number of picks (the collection “time”), i is an index over all coupons, and lambda is the probability of collecting that coupon at each pick. The inner term is the Poisson probability that at least one of each coupon has been collected, as one minus the probability of zero occurrences. The outer term is the probability that the current number of picks is not sufficient, meaning that the total number of picks required for a full collection is at least that large. In principle, knowing the probabilities for each of the coupons, using numerical integration, such as the quadrature method[14], can derive the total number of picks required. However, as the number of coupons increases, the numerical integration becomes impractical, requiring many evaluations of the inner term.

In addition to the expected value, one might want to know the standard deviation, in order to determine the number of samples required to see every coupon with some level of confidence (i.e. 95% confidence). This can be particularly difficult because the number of samples for a full collection may be much larger than that of a partial collection; for example if there are some rare members within the collection. Therefore, it would be useful to derive an expression for the number of samples required to get a partial collection (e.g. to see 95% of the coupons in the full collection). We describe below our approach to estimating both the standard deviation for the total collection as well as the number of samples needed for partial collections (e.g. 95% of full collection). These components together provide an”oversampling metric” that concisely conveys library quality and approximates screening scope.

## Methods

### Library sequencing preparation

The sample of the library, with length 717 bp, was prepared for “shotgun” sequencing on a high-throughput Illumina^®^ MiSeq sequencer. For each sample, 3.75 ng of library DNA was prepared in a 50 μl reaction with 0.5 μl of Illumina^®^ Nextera and 25 ul TD buffer at 55°C for 5 minutes. This reaction was then purified using a 0.6x SPRI magnetic bead-based purification. The resulting material appears on a gel as a smear of DNA with varying length between ~100–717 bp. The prepared DNA was used as template in a 50 μl index PCR with NEBNext^®^ master mix and Illumina^®^ indexing primers; and re-purified using magnetic bead-based purification. In final preparation, each sample was diluted to 7 pM, before running on an Illumina^®^ MiSeq sequencer using reagents from an Illumina^®^ MiSeq Reagent Kit V2, to produce 150 base paired-end reads. The reference sequence of the library used for this study appears in the supporting information (S2b Fig).

### Sequence alignment

We developed a proprietary method for read mapping called the “graphaligner”, and compared it with two standard methods: (1) an implementation of Smith-Waterman alignment[15] and (2) Bowtie 2, an aligner based on the Burrows-Wheeler transform[16].

The graphaligner builds a finite state machine (FSM) for each library reference [17]. Because each variant library is a collection of very similar sequences, all of the sequences that comprise the library can be expressed effectively in a compressed reference format as a regular expression. The output regular expression has no loops and alternatives are of fixed length, simplifying its interpretation. The alignment of a read to the reference uses the states of the resulting FSM for graphaligner alignment instead of the reference base positions used for Smith-Waterman alignment.

**Fig 1:**
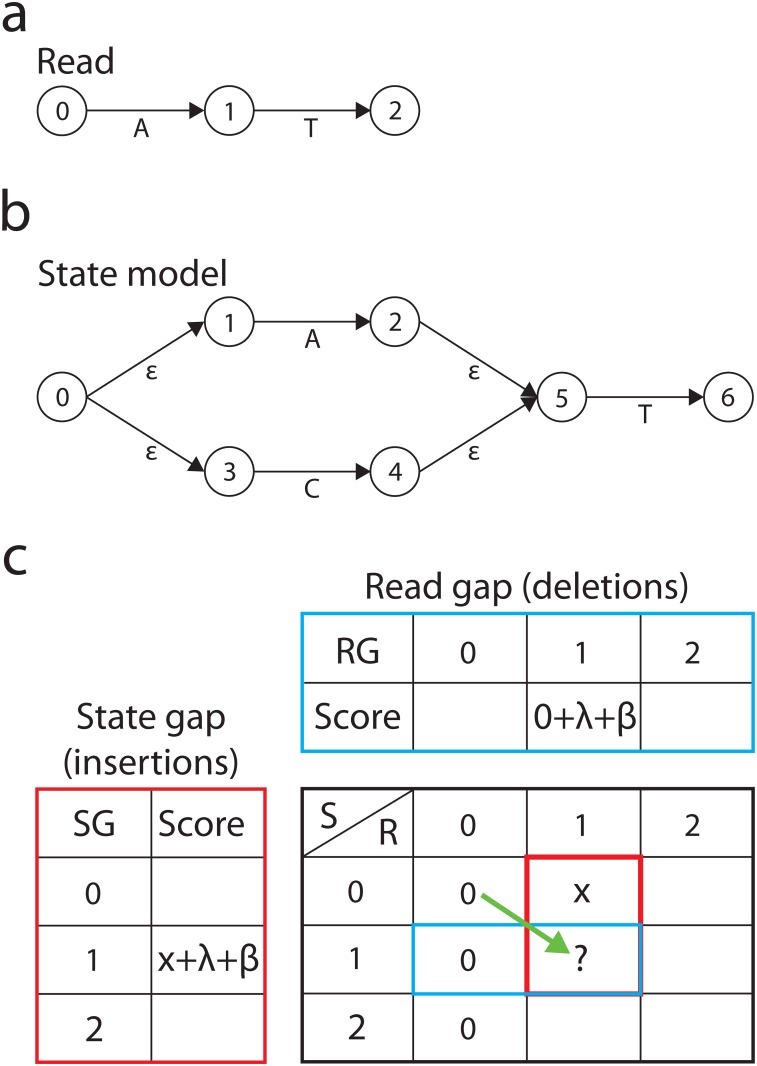
Example of the graphaligner FSM and scoring matrix. a. A theoretical next-generation sequencing read is shown, where the numbers represent sequential positional reads and the letters represent the nucleotides read at those positions. b. The state model is a representation of the FSM for a reference where the numbers represent the state of the model, the arrows are the transitions between the states, and the letters represent possible states, with ε representing an empty state indicating a deletion or an insertion of various length. c. Scoring matrices for the state model. The blue outlined box shows the scoring for a read gap (deletion) where RG is the incrementally read bases of the sequencing read, the 0 represents the initial state of the scoring matrix (subsequent scorings would use x which represents the previous state), λ is the gap opening penalty, and β is the gap extension penalty. The red outlined box shows the scoring for a state gap (insertion) where SG is the current state, x represents the score from the previous state, λ is the gap opening penalty, and β is the gap extension penalty. The black outlined box is the scoring matrix where R is the incrementally read bases of the sequencing read, S is the current state, x represents the score from the previous state, the “?” is the current score to be determined and the green arrow represents a transition for a match or mismatch (substitution) between the read and the state of the model.

The graphaligner can process alignments as local or global alignments based on the initialization and usage of the dynamic programming matrix. As with the Gotoh modification to Smith-Waterman alignment, the graphaligner allows gaps in either the reference or the read with affine gap penalties[18, 19]. Tracking of the best traversal through the FSM is complex as each state may have multiple predecessors; the maximum score from any predecessor is maintained in the dynamic programming matrix. As with other alignment methods such as Smith-Waterman, the graphaligner guarantees that there is no better score than the alignment it found, but it does not guarantee that the presented alignment is the sole alignment with the maximum score. Fig 1 shows a graphical representation of how the graphaligner functions. The state model for the graphaligner allows for variant positions where the model can be traversed through one option or another, e.g. model states 1–2 compared to states 3–4 (Fig 1b). This method allows for each state in the matrix to have multiple predecessors and therefore there are multiple paths through the FSM that are considered before a determination of a match between a read and a particular instance of the state model is made.

To evaluate the graphaligner performance, we implemented a Smith-Waterman routine with flexible scoring parameters and affine gap penalties. We also implemented Bowtie version 2.2.5 with local alignment and affine gap penalties; requiring that paired reads map to the same reference. Errors that are present in both reads of a paired-end read are considered to be "confirmed" and assigned to the variant library. Errors that are present in only one of the reads of a paired-end read are "unconfirmed" and assigned to the sequencer as a sequencing error. The overall sequencing error rate is within the normal ranges for the Illumina^®^ MiSeq instrument.

### Simulations of library screening

Simulations were performed to determine the effect of fidelity, diversity, and representation on library screening. Variant distributions were created using the uniform probability perturbed by normal random variants scaled by various fractions of the uniform probability. To prevent very small probabilities from skewing the result, we limited probabilities to at least 1/100 of the uniform probability, and measured the actual standard deviation of the resulting probabilities.

For sets of probabilities (typically 1000 variants), we computed the theoretical expected number of picks (the amount of screening required) for a full collection of library members using numerical integration from 0 to t_u_[20], which is defined as:

$$2*\;-\text{ln}\left(\text{pmin}\;*10^{-7}\right)/\text{pmin}$$

where pmin is the smallest of the item probabilities. Numerical integration often requires a reasonable limit for the integration to function properly, and we used the value in the expression above as the upper end of the range. This ensures that the range of integration is large enough such that the smallest probability component of the inside product of the theoretical expectation is close to 1, i.e. that one has almost assuredly seen at least one of the uncommon library members. The theoretical estimates—which only give the value for a full collection—were compared with simulations to verify that the simulations resulted in estimates reasonably close to the expected value (data not shown).

Library screening picks were simulated using a binary search on a probability sum structure. Picks were carried out until all members were collected, using a bit vector and global count to record whether each member has been collected. Alternatively, picks were limited to an input count to report a fraction of members collected. Full and partial collections were simulated with a large number of iterations (typically 1000) to measure the average and standard deviation of the collection picks.

Factored distribution simulations were run on a variant library made up of 4 regions with 3 options and 4 regions with 5 options, for a total of 50,625 different specified variants (the library sequence is show in the Supporting Information S2a Fig). For each simulation, the probability distribution for each variant position was randomized and the standard deviation of the completely specified (multiplied out) distribution was computed as:

$$\frac{\sqrt[{}]{\frac{\sum_{i}^{N}{\left(p_{i}-\bar{p}\right)}^{2}}{N}}}{1/N}$$

The standard deviation was used as the metric for the variation in the fully specified distribution when comparing against results for other distributions.

### Extension of the coupon collector

The coupon collector problem has a clear combinatorial solution for uniform coupon probabilities. If the picks are considered in order, the probability of seeing a new coupon at any step (where a step is defined as a set of collected coupons) is the fraction of unseen probability mass at that step:

$$p_{i}=\;\frac{N-i}{N}$$

where N is the total number of coupons, and i is the number collected. The expected number of picks from the geometric distribution to collect any of the uncollected coupons at step i is:

$$E_{i}=\frac{1}{p_{i}}=\;\frac{N}{N-i}$$

and the sum of these expectations for all steps from 0 to N-1 is the result. Note that the values may be fractional quantities rather than integers.

The calculation is extended with non-equal probabilities by maintaining the sum of the uncollected coupon probabilities as the probability of collecting any uncollected coupon. At each step, *p*_*i*_ is the sum of the remaining uncollected coupon probabilities and E_i_ is 1/p_i_. The variance of the number of picks to see the next uncollected coupon is, again from the geometric distribution, where p is the remaining probability mass:

$$\frac{1-p}{p^{2}}$$

The variance of the sum is the sum of the variances at each step.

We consider the probabilities of uncollected coupons in order from most probable to least probable, approximating that the next most probable uncollected coupon is collected at each step. This will likely vary in any actual pick sequence. If a low probability coupon is collected earlier than a more probable coupon, this results in less sampling needed to pick the remaining coupons. Thus, our method gives an overestimate of the expected number of picks required.

The degree of this overestimate was determined by simulation of collection counts for a library of 1000 members with various standard deviations relative to the uniform probability, and comparison of this with our calculated oversampling estimate. This comparison yields the overestimate factor as a ratio of the oversampling estimate over the results of the simulations. The ratios are reported as histograms in supplemental S1 Fig.

Our extension to the coupon collector method can also be used to calculate estimates of partial collections, by stopping the collection at any number of picks. The standard deviation for the number of picks at any step can be estimated by maintaining the sum of the variances of each geometric pick. In the limit of completely uniform probabilities, where the ordering of the otherwise indistinguishable coupons does not matter, our method returns the exact correct estimates for both sample size and variance of the standard coupon collector problem.

For highly diverse variant libraries, it is impractical to directly establish reasonable input estimates of the complete set of library probabilities. Instead, we estimate the final probabilities of members from a product of the probabilities at each region, assuming each region is independent. To compress the size of the set of probabilities, we bin approximate values together with an occurrence count of the bin, while maintaining the exponent / magnitude. The results can be entered into a hash table, where the resulting keys may be sorted and the largest values iterated over first.

## Results

The standard metrics for library qualification are diversity, representation of variants, and fidelity. All of these metrics are factors that determine the amount of sampling required to cover a desired fraction of the library in a screen. Since this sampling amount is greater than the size of the library (i.e. greater than the number of library members) to achieve close to complete coverage of the library, it is referred to as the oversampling metric. Ultimately, an oversampling metric provides variant library users with a meaningful and practical means to evaluate library quality.

**Fig 2:**
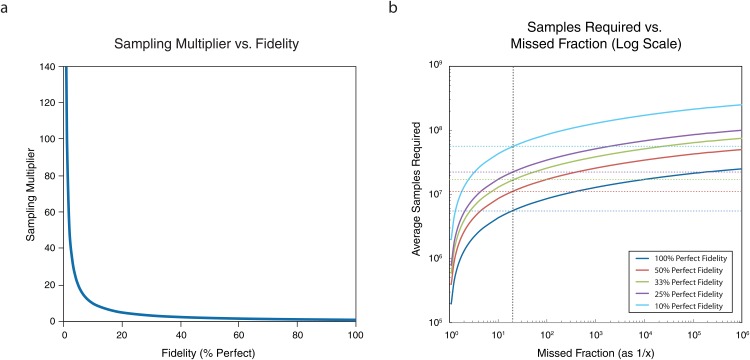
Simulations to show the impact of fidelity on the sampling multiplier needed for a library screen. **a.** *Sampling multiplier vs fidelity*. The sampling multiplier, magnitude above the size of the library that a researcher should sample, is plotted against the percentage of error-free members of a library population (% perfect). The multiplier is a simple function f(x) = 1/x, which increases rapidly with a percentage of perfect molecules < 20%. **b.** *Average samples required vs*. *missed fraction of library members*. The average number of simulated samples required to cover a certain fraction of a 2 x 10^6^ library is plotted against the missed fraction on a log scale, expressed as 1/x of powers of ten. The vertical dotted black line indicates the 95% collection position on the x-axis. Each line represents the average sampling required for libraries with the indicated fidelity: (dark blue) 100%, (red) 50%, (green) 33%, (purple) 25%, and (light blue) 10% fidelity. The corresponding horizontal dotted lines indicate the average samples required for each level of fidelity to cover 95% of all possible variants in a screen.

As the diversity of variant libraries increases, representation becomes impractical to measure directly. Calculation of fidelity remains tractable; but fidelity has less impact on the oversampling metric for diverse libraries relative to representation. As shown in Fig 2a, the sampling multiplier (magnitude above the size of the library that a researcher should sample) rises sharply due to fidelity (% perfect) only when the fidelity is poor, i.e. <20%. Simulations of a 2x10^6^ library at various levels of fidelity were performed to show the effect on the amount of sampling required to cover the library across a range of desired coverage (Fig 2b). To see 95% of all variants at a fidelity of 25% perfect requires only a 10x oversampling of the size of the library. Typically fidelity ranges for DNA libraries are observed to be within a narrow 3-fold range (e.g. 30% to 90%)[21, 22].

**Fig 3:**
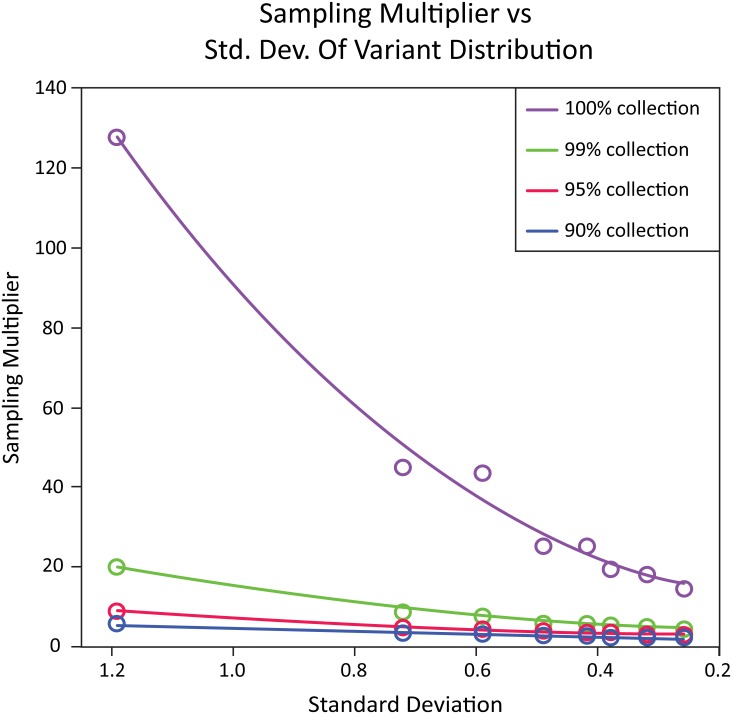
The sampling multiplier (oversampling) needed in a screen of a library for full and partial collections of possible variants and the impact of the deviation from a uniform distribution of variant member counts. The sampling multiplier is plotted against the standard deviation from the uniform distribution of variant member counts for simulations of a test library with ~50K variants. High standard deviation from uniform variant distribution indicates uneven representation. The circles represent the oversampling required for collection of various percentages of library variants: (blue) 90%, (red) 95%, (green) 99%, and (purple) 100%. The corresponding solid lines are best fit lines to indicate the trends of the data points.

Uneven representation has an even more significant impact on oversampling as library diversity increases and when more complete sampling coverage of the library is required. Results from simulations of libraries with deviations from a uniform distribution are shown in Fig 3. The sampling multiplier is the amount of sampling over the library size one would need on average to cover the desired percentage of variants (for example, the purple line in Fig 3 is for 100% variant coverage). The variance of the sampling multiplier increases with larger variance of the probabilities and the completeness of the collection. Note that these simulations have the departures from uniform set to be small and fairly normal, approximately Gaussian. Any members with extremely small probabilities will require many samples to cover, and the sampling multiplier rises sharply with these occurrences, as does its variance. In addition, the sampling multiplier increases sharply for >95% variant coverage (as shown in Fig 3).

**Fig 4:**
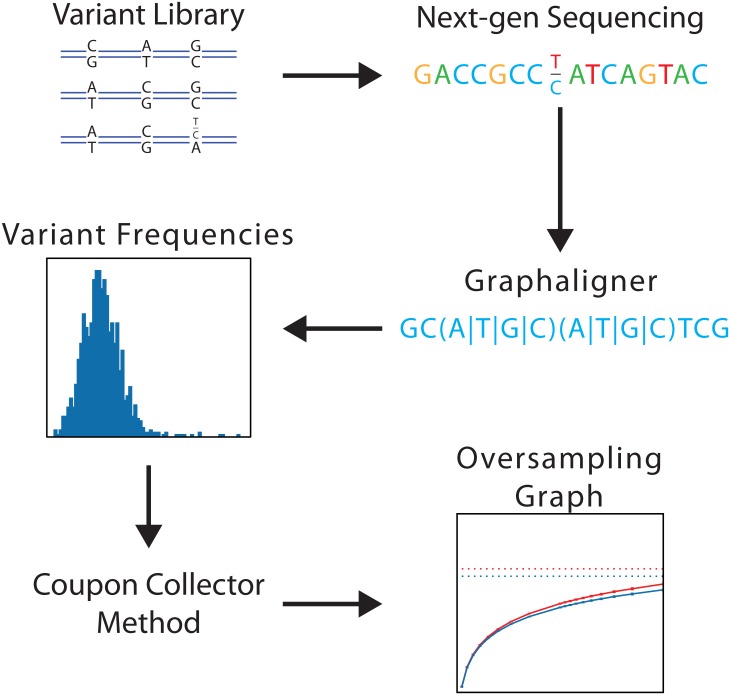
The method to determine an estimate of oversampling required for screening a DNA variant library using NGS data. A highly variant library is loaded onto a next generation sequencing instrument. An alignment of the library sequence is carried out using our graphaligner alignment method. Frequency distributions of all variants are found. The coupon collector method estimate is adjusted for an overestimate factor and is then used to generate an oversampling graph specific for this library. The oversampling metric is determined by the oversampling graph for a targeted percentage of the library to be screened.

We have developed a process (Fig 4) for establishing an oversampling metric for variant libraries. The process enables the calculation of variant representation within highly diverse variant libraries (implicitly measuring the diversity). In addition, the representation measurements are integrated with fidelity measurements to generate a comprehensive view of the oversampling required to cover these libraries at any desired percentage of coverage. The first step to characterize a highly diverse DNA library is to sequence the fragmented library on a high throughput next-generation sequencer to generate millions of paired-end reads. The reads are aligned with a novel algorithm, called the graphaligner, which efficiently identifies the variant choices for each read. In addition, fidelity is calculated from the per-base error rate of the library sequences. The per-base error rate is calculated by measuring the number of single and multiple nucleotide deletions/insertions, and individual substitutions found in the sequencing data, and dividing the sum of those errors by the total number of bases read in the sequencing run. An example reference sequence along with mock sequencing reads and the resulting per-base error calculation are shown in the supporting information (S2b Fig). The relative frequencies of each of the variant positions from the set of aligned reads are calculated, measuring the representation at each variant position. The representation and fidelity data are used in an extension to the coupon collector method to generate the oversampling graph, which in turn expresses the oversampling metric (average samples required/size of library population) for a range of missed fractions of the library.

One important factor when calculating the oversampling metric is mapping the NGS sequencing reads, and the ability to accurately align the many sequencing reads to the reference in a reasonable amount of time. Standard alignment tools, such as Smith-Waterman alignment, and Burrows-Wheeler transform based alignment (Bowtie 2, etc.) are insufficient for processing the many similar reference sequences of DNA variant libraries.

We have attempted to use two standard alignment tools to process the references of a 65 bp test library (the sequence appears in Supporting Information S2a Fig) with 50,625 distinct members (Table 1). Both standard tools, Bowtie 2 and Smith-Waterman, require the alignment against all library member sequences. This becomes unmanageable as the number of library members increases. For example, a 1 kbp library with 3 x 10^9^ members would have a reference sequence space roughly a thousand times the size of the human genome.

**Table 1:** Statistics of alignment with three different sequence alignment tools. Illumina^®^ MiSeq reads were aligned with three different statistical alignment methods to compare ease of use and computational time. Reads for a variant library 65 bp in length with 50,625 members were processed using the graphaligner, Smith-Waterman alignment, and Bowtie 2 (Bt2). The number of references N, mapping rate, map time to process 1 million reads, the percent suboptimal mappings and the relative speed are shown. The build times for the FSM in the graphaligner and the Bowtie 2 FM-index (an index of the full-text substrings from the regular expression representation of the library, which is compressed and allows for fast substring queries) [23] are included in the read rates and are negligible. We have estimated the metrics for 50,625 references for Smith-Waterman from a run with 1000 references, due to the large computational time for 50,625 references.

Method	Number of References	Mapping rate (reads / sec)	Map Time/1 Million Reads (minutes)	Percent Suboptimal mappings	Relative speed
Graphaligner	1*	91.84	181.47	0	1
Smith-Waterman	N(est. 50625)	0.012	1386536.5	0	0.0001
Bt2 very fast local	N (= 50625)	1280.69	13.01	94	13.94
Bt2 very sensitive local	N (= 50625)	242.48	68.73	70	2.64

* The graphaligner reference represents all 50,625 different variant sequences in a single compact regular expression format.

Table 1 indicates that the computational time of the alignment increases dramatically for the Smith-Waterman aligner, ultimately rendering it useless for processing the millions of reads generated by a standard high throughput next-generation sequencer. For the modest size of the test library reference (50,625), Smith-Waterman alignment would require 23,108 hours of computation to map 1 million reads. However, Bowtie 2 proved to be more resilient for computation time. It is quite fast, processing all of the reads in 0.2 hours with the very fast local settings and 1.14 hours with the very sensitive local settings (the settings used for Bowtie 2 appear in the Supporting Information).

We also tested the optimality of the differing algorithm mappings from graphaligner and Bowtie 2 by scoring those mappings with Smith-Waterman. Bowtie 2 explicitly disclaims the ability to guarantee the best scoring alignment [24] due to its heuristic algorithm. We confirmed that Bowtie 2 returns incorrect alignments for variant library sequence input and therefore incorrect oversampling metric estimates. The very fast local Bowtie 2 settings have a suboptimal mapping when compared to Smith-Waterman in 94% of the mappings, while the very sensitive local settings have a suboptimal mapping of 70%. We have included FASTA files with the Supporting Information files S1 and S2 Texts showing alignments for Bowtie 2 and graphaligner where those tools differ in mapping. These alignments clearly demonstrate the suboptimal mappings from Bowtie 2 in that there are far more mismatches in the Bowtie 2 file for the same sequencing reads when compared to graphaligner.

To solve the reference space, suboptimal mapping and computation time issues, we developed a proprietary aligner, called graphaligner, which effectively processes all possible variant references using a finite state machine (as described in the methods). The graphaligner was able to process the 1 million test library reads against this single reference in only 3 hours, with equivalent accuracy to Smith-Waterman alignment (i.e. no suboptimal mappings). The graphaligner is able to find a consensus sequence interpretation from the reads and the relevant alignments.

**Fig 5:**
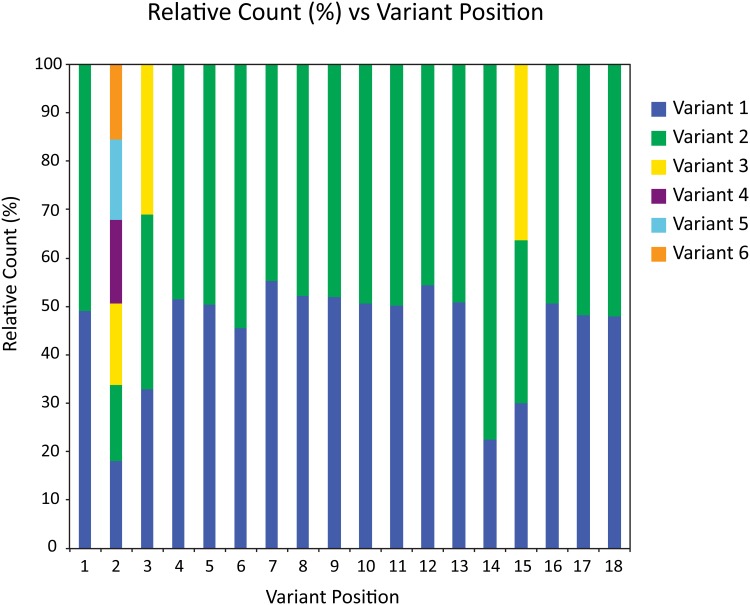
Relative count (percentage) of variants at each variant position. The y-axis represents the normalized relative percentage of the possible variants at each variant position. The x-axis represents the positions of variation in sequential order along the length of the library sequence. The number of colors in a bar represents the number of possible variants at that variant position in the library.

The variant position calls, once identified by the graphaligner, are used to count the frequency of variants amongst the library members. Fig 5 shows the normalized counts of all possible variants at the 18 variant positions of a test library sequenced and aligned with the graphaligner. The number of times that a variant was seen is counted and compared to the other possibilities at that position. It is clear from the data that this test library has an evenly distributed number of variants at most positions. Variant position 14, however, is skewed in that we see fewer of the first variant relative to the second variant, with a ratio of approximately 2.5 to 1 (Fig 5). The disproportionate ratio between variant possibilities such as that at position 14 in this test library (Fig 5) is the driving force in increasing the oversampling required to see all possible variants in a library screen.

**Fig 6:**
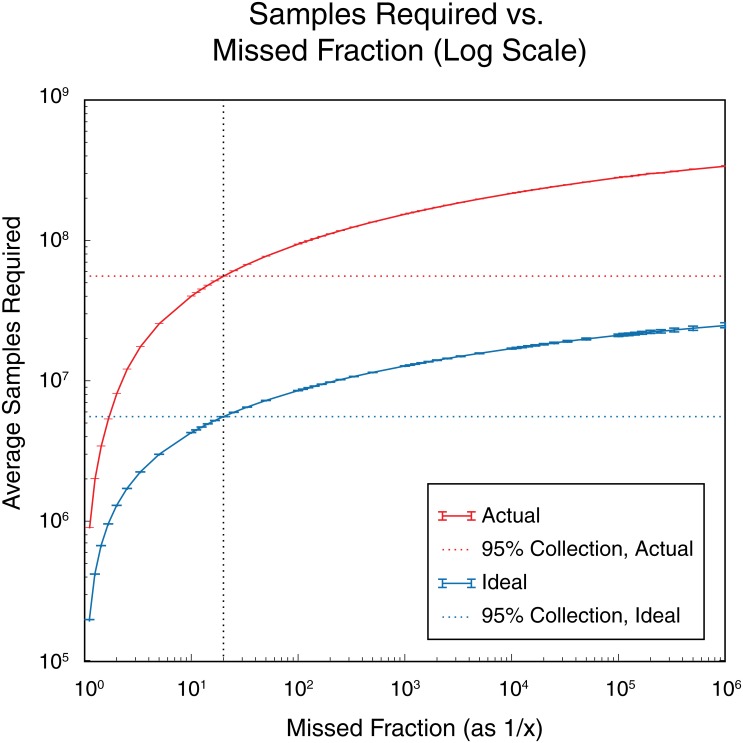
Oversampling graph. Average samples required vs. missed fraction (1/x) of variants for a variant library with overall diversity of 2x10^6^ possible variants. The blue solid line represents the samples required vs. the missed fraction for the library with an ideal, flat distribution of all possible variants and perfect fidelity for the library. The red solid line represents the samples required vs. the missed fraction for the library with the actual measured representation and fidelity of all variants in the library. The blue dotted line represents the average samples required to collect 95% of all possible variants with an ideal distribution. The red dotted line is the average number of samples to collect 95% of all possible variants with measured distribution of variants in the actual library. The oversampling metric would be the average sampling required/size of the library population to screen a particular missed fraction of the library population.

When using these variant counts in calculating the oversampling estimate, the assumption that the most prevalent variants would appear first is a stringent constraint that leads to an overestimate of the required sampling. Supplemental S1 Fig shows the overestimate factor for simulated data distributions; the number of samples in the overestimate factor bins determines the frequency shown on the y-axis. The x-axis is the overestimate factor, i.e. the ratio of the coupon collector method oversampling estimate, over the exact oversampling required for the known simulated data distributions. Our method provides an upper bound that overestimates the true theoretical value of the oversampling required, by a factor of 1.5–2.0 (S1 Fig). Accordingly, we take this overestimate factor into account and divide by 1.5 to produce the oversampling graph curves (Fig 6).

The result of the process as described above is the oversampling graph in Fig 6. The oversampling metric is the amount of oversampling required for a chosen missed fraction (percentage of library coverage) as indicated by this graph. The solid blue line shows the required samples at different levels of missed fractions of the total library population (as 1/ missed fraction) for our 2x10^6^ test library assuming 100% fidelity and perfectly flat distribution for all variants. This blue line is a theoretical curve calculated for the number of variants in the library and requires no measurements of the fidelity or the representation of variants of the actual library. The blue line is a curve that can be generated from *in silico* sequence data before any attempt has been made to create the library in the laboratory. The solid red line is a curve calculated by taking into account the measured values of fidelity and representation from the sequencing data of the existing library. This red line will always indicate a higher level of required samples than the blue line, as there will be some level of error in the fidelity and some imperfection in the distribution of variants for any physical library created in a laboratory. The corresponding dotted lines in the graph represent the oversampling required to collect 95% of all possible variant species in the library. The vertical, black dotted line indicates the 95% collection point where it intersects the red and blue curves. The horizontal blue dotted line indicates the ideal samples required to see 95% of all possible variants if the theoretically ideal library had no errors or imperfections in the distribution of the variants. In this case, if it were possible to sample a theoretical library 5.5x10^6^ samples would be needed in a screen to see a 95% collection, indicating an ideal oversampling metric of 2.75 times the size of the library. The horizontal red dotted line indicates the actual samples required to see 95% of all possible variants for the actual library created in the laboratory. In this case, 5.5x10^7^ samples are needed in a screen to see a 95% collection, which is an actual oversampling metric of 27.5 times the size of our 2x10^6^ library. The actual oversampling metric (rather than the ideal oversampling metric) is meant to better guide the researcher in determining the level of screening that should be carried out for each particular library.

## Discussion

When creating a diverse DNA variant library, whether through synthesis or other means, researchers hope to approximate the intended design as accurately as possible. There are two main concerns when measuring the quality of a library to be screened. The first is the presence of unintended sequences in the library, caused by nucleotide substitutions, deletions, insertions, or rearrangements, often referred to as infidelity. Second, it is important to measure the presence of different intended variants in the library (diversity) and the relative frequency of these intended variants (representation).

Currently, library specifications are often limited to measurements of diversity and fidelity of non-variant regions. Measuring fidelity of only the non-variant regions is straightforward as there are well-tested methods such as Bowtie 2 for utilizing NGS data to build consensus sequences and determine the error rate. Variant regions pose a greater challenge as the number of reference sequences grows, and existent alignment strategies either fail in correctly mapping sequencing reads or are too slow to be practical. As a result, often there is no attempt to assess fidelity of the variant regions. Fidelity measurements between the variant and non-variant regions of a library may differ depending upon the assembly and error correction methods used to create the library.

A library can have full diversity and high fidelity while having poor representation. For example, a library may contain each possible variant but with an uneven frequency of variant library members. Also, the library population may be too large to sufficiently determine a distribution of variant possibilities even with the high number of reads generated by current next-generation sequencing instruments. Measuring representation accurately is one of the main challenges to determining the oversampling metric for a library screen.

Without the availability of an accurate oversampling metric, many researchers use a guideline of screening 10x the number of samples relative to the size of the library [25]. When collecting 95% of all possible variants, this rule-of-thumb works well if the diversity of the library is low and the error rate and deviation from a uniform distribution of the library elements is small. Advances in synthetic biology have eased the creation of highly diverse variant libraries. The 10x oversampling rule-of-thumb approach is limiting when the diversity is high, the fidelity is low, or the representation of variants is uneven. Limiting researchers to a low diversity library, or screening only a small percentage of a high diversity library reduces the likelihood of discovering improved qualities of interest via a library screen.

Diversity, representation, and fidelity measurements can indicate how effective a screen will be when using a particular level of oversampling. This is a significant consideration when the massive oversampling required for complete coverage of all library members is not practical. The oversampling graph communicates the coverage to sampling function compactly. It also shows the library quality as the difference between the functions for the ideal case and the actual measured case.

To achieve the oversampling metric we have demonstrated the graphaligner is a useful approach to mapping reads for a given complex combinatorial reference. The run time is of order L*M where M is the number of states of the reference finite state machine—roughly the total number of nucleotides across variants—and L is the length of the read in bases.

While Smith-Waterman can generate an optimal alignment for NGS reads and Bowtie 2 can be efficient for large numbers of variant sequences, both algorithms become inconvenient when it is necessary to expand the definition of a diverse library into all explicit sequences. Smith-Waterman fails to align large sets of library sequence reads within a reasonable computational run time. The run time of Smith-Waterman is of order L*N where L is length of read, and N is number of references (number of variants in the library). Bowtie 2 fails to find the optimal alignment when using highly similar references, such as those of a variant DNA library, together with short sequencing reads.

We have shown that our extension of the concept of the coupon collector problem offers a unique and effective way to represent screening of a highly variant DNA library. Our method can generate the oversampling metric for a large library with reasonable computational run time.

Our results indicate the need for oversampling in screening variant libraries. Some multiple of the library size is necessary even in ideal cases where there are completely uniform distributions. Oversampling can be small for synthetic DNA variant libraries, approximately 10x the library size, as long as the departure from uniform and the error rate are not too great. However, it is important to consider whether the level of oversampling is sufficient for the desired coverage of diverse variant DNA libraries. The oversampling graph and corresponding oversampling metric give researchers a concise way to convey the important aspects of the library quality and oversampling requirements to use effectively in a screen.

## Supporting Information

S1 Fig S1_overestimate_factors.eps. Histograms of overestimate factor frequency for various randomized distributions. These overestimate factors were calculated for a 90%, 95%, 99%, and 100% collection by comparing the oversampling estimate calculated using our method, to the required oversampling based on known probabilities for a library with 50,625 distinct variant members (S2a Fig) at a standard deviation of 0.3, 0.5 and 0.6 from a uniform distribution of variant counts. The library was tested at each standard deviation/collection level and the data are binned by overestimate factor range. Simulations of libraries with 100 and 10,000 distinct variant members give similar histograms (data not shown).
(EPS)

S2 Fig S2_sequences_alignments.docx. a. The Sequence used for Table 1 of the main text and S1 Fig. This is a 65 bp sequence with 50,625 possible variants. b. The sequence used for Figs 5 and 6 in the main text is presented as nucleotides for the non-variant positions, while the variant positions are indicated as amino acids. The brackets indicate a single position with all possible amino acid variants at that position. The particular codons used for the amino acids are indicated above. c. In the above example, there are six mock sequencing reads shown with 7 total errors (deletions represented with a “-“, and substitutions in bold red letters). The total bases read in this case is 85 as deletions are not counted. To get the per-base error rate we divide the errors (7) by the total reads (85) for a per-base error rate of 0.08.
(DOCX)

S1 Text S3_bowtie2_setting.docx. Bowtie 2 Settings.
(DOCX)

S2 Text S4_bowtie2_sw_fwd_alignments.txt. Alignment text illustration files which show alignments for reads where the graphaligner and bowtie 2 called the reads as mapping to differing references. The alignments here are Smith-Waterman re-alignments of each read to the reference called by bowtie 2.
(TXT)

S3 Text S5_graphaligner_sw_fwd_alignments.txt. Alignment text illustration files which show alignments for reads where the graphaligner and bowtie 2 called the reads as mapping to differing references. The alignments here are Smith-Waterman re-alignments of each read to the reference called by the graphaligner.
(TXT)

S4 Text S6_reads_fwd_10ksample.fa. FASTA files with a sample of paired reads. Each file contains either the 'fwd' (R1) reads.
(FA) Click here for additional data file.

S5 Text S7_reads_rev_10ksample.fa. FASTA files with a sample of paired reads. Each file contains either the 'rev' (R2) reads.
(FA) Click here for additional data file.

S6 Text S8_reference_display.txt. A text description showing the reference backbone sequence and the variant location codons making up the 50,625 combinatorial possible variants.
(TXT)

S7 Text S9_reference_variants.fa. A FASTA file with all 50,625 variant sequences possible in the variant library.
(FA) Click here for additional data file.

## References

[1] Bradley LH. High-quality combinatorial protein libraries using the binary patterning approach. Methods Mol Biol. 2014;1216:117–28. 10.1007/978-1-4939-1486-9_6 25213413

[2] Gan R, Jewett MC. Evolution of translation initiation sequences using in vitro yeast ribosome display. Biotechnol Bioeng. 2016. 10.1002/bit.25933 26757179

[3] Kosuri S, Eroshenko N, Leproust EM, Super M, Way J, Li JB, et al Scalable gene synthesis by selective amplification of DNA pools from high-fidelity microchips. Nat Biotechnol. 2010;28(12):1295–9. 10.1038/nbt.1716 21113165 PMC3139991

[4] LeProust EM, Peck BJ, Spirin K, McCuen HB, Moore B, Namsaraev E, et al Synthesis of high-quality libraries of long (150mer) oligonucleotides by a novel depurination controlled process. Nucleic Acids Res. 2010;38(8):2522–40. 10.1093/nar/gkq163 20308161 PMC2860131

[5] Poust S, Hagen A, Katz L, Keasling JD. Narrowing the gap between the promise and reality of polyketide synthases as a synthetic biology platform. Curr Opin Biotechnol. 2014;30:32–9. 10.1016/j.copbio.2014.04.011 24816568

[6] Hoebenreich S, Zilly FE, Acevedo-Rocha CG, Zilly M, Reetz MT. Speeding up directed evolution: Combining the advantages of solid-phase combinatorial gene synthesis with statistically guided reduction of screening effort. ACS Synth Biol. 2015;4(3):317–31. 10.1021/sb5002399 24921161

[7] Kosuri S, Goodman DB, Cambray G, Mutalik VK, Gao Y, Arkin AP, et al Composability of regulatory sequences controlling transcription and translation in Escherichia coli. Proc Natl Acad Sci U S A. 2013;110(34):14024–9. 10.1073/pnas.1301301110 23924614 PMC3752251

[8] Patwardhan RP, Lee C, Litvin O, Young DL, Pe'er D, Shendure J. High-resolution analysis of DNA regulatory elements by synthetic saturation mutagenesis. Nat Biotechnol. 2009;27(12):1173–5. 10.1038/nbt.1589 19915551 PMC2849652

[9] Quan J, Saaem I, Tang N, Ma S, Negre N, Gong H, et al Parallel on-chip gene synthesis and application to optimization of protein expression. Nat Biotechnol. 2011;29(5):449–52. 10.1038/nbt.1847 21516083

[10] Townsend S, Fennell BJ, Apgar JR, Lambert M, McDonnell B, Grant J, et al Augmented Binary Substitution: Single-pass CDR germ-lining and stabilization of therapeutic antibodies. Proc Natl Acad Sci U S A. 2015;112(50):15354–9. 10.1073/pnas.1510944112 26621728 PMC4687607

[11] Motwani R, Raghavan P. Randomized algorithms. Cambridge; New York: Cambridge University Press; 1995 xiv, 476 p. p.

[12] Flajolet P, Gardy D., Thimonier L. Birthday paradox, coupon collectors, caching algorithms and self-organizing search. Discrete Applied Mathematics. 1992;39(3):207–89.

[13] Ferrante M, Saltalamacchia M. The Coupon Collector's Problem. MATerials MATemàtics. 2014;2:1–35.

[14] Davis PJ, Rabinowitz P. Methods of numerical integration. New York,: Academic Press; 1975 xii, 459 p. p.

[15] Smith TF, Waterman MS. Identification of common molecular subsequences. J Mol Biol. 1981;147(1):195–7. 7265238 10.1016/0022-2836(81)90087-5

[16] Langmead B, Salzberg SL. Fast gapped-read alignment with Bowtie 2. Nat Methods. 2012;9(4):357–9. 10.1038/nmeth.1923 22388286 PMC3322381

[17] Vidal E, Thollard F, de la Higuera C, Casacuberta F, Carrasco RC. Probabilistic finite-state machines—part I. IEEE Trans Pattern Anal Mach Intell. 2005;27(7):1013–25. 10.1109/TPAMI.2005.147 16013750

[18] Gotoh O. An improved algorithm for matching biological sequences. J Mol Biol. 1982;162(3):705–8. 7166760 10.1016/0022-2836(82)90398-9

[19] Altschul SF, Erickson BW. Optimal sequence alignment using affine gap costs. Bull Math Biol. 1986;48(5–6):603–16. 3580642 10.1007/BF02462326

[20] Boneh A, Hofri M. The coupon-collector problem revisited—a survey of engineering problems and computational methods. Communications in Statistics PartC: Stochastic Models. 1996;13(1):39–66.

[21] Ma S, Saaem I, Tian J. Error correction in gene synthesis technology. Trends Biotechnol. 2012;30(3):147–54. 10.1016/j.tibtech.2011.10.002 22209624 PMC3933390

[22] Carr PA, Park JS, Lee YJ, Yu T, Zhang S, Jacobson JM. Protein-mediated error correction for de novo DNA synthesis. Nucleic Acids Res. 2004;32(20):e162 10.1093/nar/gnh160 15561997 PMC534640

[23] Ferragina P, Manzini G. Opportunistic Data Structures with Applications. Proceedings of the 41st Annual Symposium on Foundations of Computer Science. 2000:390–8.

[24] Langmead B. Bowtie 2 Manual 2016. http://bowtie-bio.sourceforge.net/bowtie2/manual.shtml.

[25] Arndt KM, Muüller KM. Protein engineering protocols. Totowa, N.J: Humana Press; 2007 xi, 312 p. p.

