# Supplementary material for: Determination of a Screening Metric for High Diversity DNA Libraries
Source: PLoS One. 2016 Dec 8;11(12):e0167088. doi: 10.1371/journal.pone.0167088 (PMC5145166; doi:10.1371/journal.pone.0167088)
Supplement: S2 Fig — a. The Sequence used for Table 1 of the main text and S1 Fig. This is a 65 bp sequence with 50,625 possible variants. b. The sequence used for Figs 5 and 6 in the main text is presented as nucleotides for the non-variant positions, while the variant positions are indicated as amino acids. The brackets indicate a single position with all possible amino acid variants at that position. The particular codons used for the amino acids are indicated above. c. In the above example, there are six mock sequencing reads shown with 7 total errors (deletions represented with a “-“, and substitutions in bold red letters). The total bases read in this case is 85 as deletions are not counted. To get the per-base error rate we divide the errors (7) by the total reads (85) for a per-base error rate of 0.08. (DOCX) [file pone.0167088.s002.docx]

## S2 Fig

**a.**

**Sequence of variant library used for Table 1 and Fig S1**

AAGCTCGCCTGTATCTTAGTCGGGGTTGCATCAAGCATATCGTCGGGTGCGGGGAAGCCAACATC

GAG GAT ATC TTC TAC ATC AAT GGC

GTC GCG AGG ATC GCT AAT ACA AGC

CCC TAT CCC TTG

AAA CCC CTA CCG

## b.

## Sequence of variant library used for Fig. 5 and 6 of the main text

ATGAGTAAAGGAGAAGAACTTTTCACTGGAGTTGTCCCAATTCTTGTTGAATTAGATGGTGATGTTAATGGGCACAAATTTTCTGTCAGTGGAGAGGGTGAAGGTGATGCAACATACGGAAAACTTACCCTTAAATTTATTTGCACTACTGGAAAACTACCTGTTCCATGGCCAACACTTGTCACTACT[FL][STGCLA][YFH][GT][VL][QM][CV]TTT[SA]AGATACCCAGATCATATGAAA[QR]CATGACTTTTTCAAGAGTGCCATGCCCGAAGGTTATGTACAGGAAAGAACTATA[FS]TTCAAAGATGACGGGAACTACAAGACACGTGCTGAAGTCAAGTTTGAAGGTGATACCCTTGTTAATAGAATCGAGTTAAAAGGTATTGATTTTAAAGAAGATGGAAACATTCTTGGACACAAATTGGAATACAAC[YF]AACTCACAC[NK]GTATACATC[MT]GCAGACAAACAAAAGAATGGAATCAAA[VA]AACTTCAAA[IVT]AGACACAACATTGAAGATGGA[SG]GTTCAACTAGCAGACCATTATCAACAAAATACTCCAATTGGCGATGGCCCTGTCCTTTTACCAGACAACCATTACCTG[SF][TI]CAATCTGCCCTTTCGAAAGATCCCAACGAAAAGAGAGACCACATGGTCCTTCTTGAGTTTGTAACAGCTGCTGGGATTACACATGGCATGGATGAACTATACAAATAA

### Codon usage

A:GAT

C:TGC

D:GAC

E:GAA

F:TTC

G:GGT

H:CAC

I:ATC

K:AAA

L:TTG

M:ATG

N:AAC

P:CCG

Q:CAG

R:CGT

S:AGC

T:ACC

V:GTT

W:TGG

Y:TAC

**c.**

## Per-base error calculation example:

Example Reference sequence:

ATGAGTAAAGGAGAAGAACTTTTCACTGGAGTTGTCCCAATTCTTGTTGAATTAGATGG

Mock Sequencing reads:

ATGAGTAA--GAGAAGA

AAGAACTTTT**AG**CTGG

ACT-TTCACTGGAGTTG

TTGTCCCAATTCTTG

ATTCTTG---AAT

TTGAA**AA**AGATGG

Example Per-base Error rate:

(2 multi-nucleotide deletion + 1 single nucleotide deletions + 4 nucleotide substitutions)/(85 reference nucleotides) = 0.08 errors/base
