# Supplementary material for: Determination of a Screening Metric for High Diversity DNA Libraries
Source: PLoS One. 2016 Dec 8;11(12):e0167088. doi: 10.1371/journal.pone.0167088 (PMC5145166; doi:10.1371/journal.pone.0167088)
Supplement: S1 Text — Bowtie 2 Settings. (DOCX) [file pone.0167088.s003.docx]

S3 Fig

## Bowtie 2 Settings

Bowtie 2 very fast local (50k references)

bowtie2 --local -x ../refs/all --np 0 --rdg 20,1 --rfg 20,1 --ma 10 --mp 8,8 --score-min G,400,0 -p 10 -U ../reads/fwd.fa -f -D 5 -R 1 -N 0 -L 25 -i S,1,2.0 -S bt2_output.sam

Bowtie 2 very sensitive local (50k references)

bowtie2 --local -x ../refs/all --np 0 --rdg 20,1 --rfg 20,1 --ma 10 --mp 8,8 --score-min G,400,0 -p 10 -U ../reads/fwd.fa -f -D 20 -R 3 -N 0 -L 20 -i S,1,0.5 -S bt2_output.sam

The very fast local and the very sensitive local Bowtie 2 settings were chosen to represent the fastest and the most sensitive preset settings for Bowtie 2 respectively. These are the standard settings that should perform most favorably when comparing the metrics of read mapping per minute and percent suboptimal mappings to those of Smith-Waterman and graphaligner.
